# AhaP, A Quorum Quenching Acylase from *Psychrobacter* sp. M9-54-1 That Attenuates *Pseudomonas aeruginosa* and *Vibrio coralliilyticus* Virulence

**DOI:** 10.3390/md19010016

**Published:** 2021-01-01

**Authors:** José Carlos Reina, Manuel Romero, Rafael Salto, Miguel Cámara, Inmaculada Llamas

**Affiliations:** 1 Department of Microbiology, Faculty of Pharmacy, Campus Universitario Cartuja s/n, University of Granada, 18071 Granada, Spain; josecreina@ugr.es; 2 National Biofilms Innovation Centre, Biodiscovery Institute and School of Life Sciences, University of Nottingham, Nottingham NG7 2RD, UK; M.Romero@nottingham.ac.uk (M.R.); Miguel.Camara@nottingham.ac.uk (M.C.); 3 Department of Biochemistry, Faculty of Pharmacy, Campus Universitario Cartuja s/n, University of Granada, 18071 Granada, Spain; rsalto@ugr.es; 4 Biomedical Research Center (CIBM), Institute of Biotechnology, University of Granada, 18100 Granada, Spain

**Keywords:** quorum quenching, acylase, *Psychrobacter*, marine habitat

## Abstract

Although *Psychrobacter* strain M9-54-1 had been previously isolated from the microbiota of holothurians and shown to degrade quorum sensing (QS) signal molecules C6 and C10-homoserine lactone (HSL), little was known about the gene responsible for this activity. In this study, we determined the whole genome sequence of this strain and found that the full 16S rRNA sequence shares 99.78–99.66% identity with *Psychrobacter pulmonis* CECT 5989^T^ and *P. faecalis* ISO-46^T^. M9-54-1, evaluated using the agar well diffusion assay method, showed high quorum quenching (QQ) activity against a wide range of synthetic *N*-acylhomoserine lactone (AHLs) at 4, 15, and 28 °C. High-performance liquid chromatography-mass-spectrometry (HPLC-MS) confirmed that QQ activity was due to an AHL-acylase. The gene encoding for QQ activity in strain M9-54-1 was identified from its genome sequence whose gene product was named AhaP. Purified AhaP degraded substituted and unsubstituted AHLs from C4- to C14-HSL. Furthermore, heterologous expression of *ahaP* in the opportunistic pathogen *Pseudomonas aeruginosa* PAO1 reduced the expression of the QS-controlled gene *lecA*, encoding for a cytotoxic galactophilic lectin and swarming motility protein. Strain M9-54-1 also reduced brine shrimp mortality caused by *Vibrio coralliilyticus* VibC-Oc-193, showing potential as a biocontrol agent in aquaculture.

## 1. Introduction

The term quorum sensing (QS), which refers to a well-known population density-dependent gene expression mechanism, was introduced for the first time by Fuqua et al. in 1994 [1]. QS enables bacteria to communicate with each other through the production, release and detection of signal molecules, also known as autoinducers. After reaching a threshold concentration in the surrounding medium, these autoinducers coordinate the expression of multiple genes, including those coding for antibiotic and exoenzyme production, as well as for biofilm formation [2,3,4]. Some of the most studied autoinducers produced by Gram-negative bacteria are *N*-acylhomoserine lactones (AHLs). The canonical AHL molecule contains a homoserine lactone ring linked to an acyl chain which can present different levels of saturation and substitution [5,6,7].

AHL-mediated QS systems control virulence gene expression in multiple pathogens, including marine, agricultural and human pathogens [8,9,10,11,12,13]. Hence, interference with QS systems has been proposed as a novel strategy to prevent or attenuate these infections [14]. This interference can be achieved using molecules that block the interaction of AHLs with their cognate signal receptors without affecting signal integrity. These molecules are generally named QS inhibitors (QSIs). Alternatively, QS signaling can be disrupted by enzymatic inactivation of the signal molecules, a mechanism known as quorum quenching (QQ), which is one of the most studied approaches used to interfere with QS-mediated regulatory mechanisms in order to control bacterial infections [15].

There are three main types of AHL-QQ enzymes: lactonases, which open the lactone ring; acylases, which cleave the AHL amide bond; and oxidoreductases, which modify the fatty acid chain whose recognition is impeded by the signal receptor [16,17]. Enzymatic degradation of AHLs has been reported in a wide range of microorganisms, including AHL- and non-AHL-producing bacteria [18], as well as in mammalian cells [19], suggesting that QS inhibitory processes play an important role in different environments, whose actual ultimate physiological function, however, remains unexplained [20].

QQ enzymes have been shown to be effective in preventing infections caused by AHL-producing bacterial pathogens in konjac [21], potato tubers [22,23,24], tobacco and cauliflower [25], and shrimps [10,26,27]. QQ enzymes have also been shown to reduce the virulence of the human opportunistic pathogen *Pseudomonas aeruginosa* in several infection models [28,29]. With regard to the potential use of different QQ enzyme types in the fight against infections, it has been proposed that acylases, whose AHL-degrading activity is irreversible, could be more effective than lactonases, whose degradation of AHLs can be reverted in acidic environments [30,31].

The marine environment is a prolific and valuable source of numerous bioactive compounds [32], including quorum sensing inhibitor (QSI) and QQ enzymes [33,34,35,36,37]. Our laboratory recently examined marine invertebrates such as sea anemones and holothurians whose microbiota are a source of antimicrobial molecules [38]. From the same collection of bacteria, several active AHL-degrading bacteria from *Stenotrophomonas maltophilia* isolates [24] and a QSI-producing strain of *Vibrio alginolyticus* [39] have been identified and characterized. In this study, we analyzed the AHL-degrading capacity of strain M9-54-1 which was isolated from a holothurian and identified as belonging to the genus *Psychrobacter*. The QQ activity of M9-54-1 was tested against a wide range of synthetic AHLs, as well as crude extracts from AHL-producing aquacultural and human pathogens. We identified the gene responsible for this activity and also purified and characterized the enzyme encoded by this gene. The ability of this QQ enzyme to interfere with the QS systems of the pathogens *Pseudomonas aeruginosa* PAO1 and *Vibrio coralliilyticus* VibC-Oc-193 was also evaluated.

## 2. Results

### 2.1. Psychrobacter sp. M9-54-1 Shows Broad AHL-Degrading Activity

**Figure 1:**
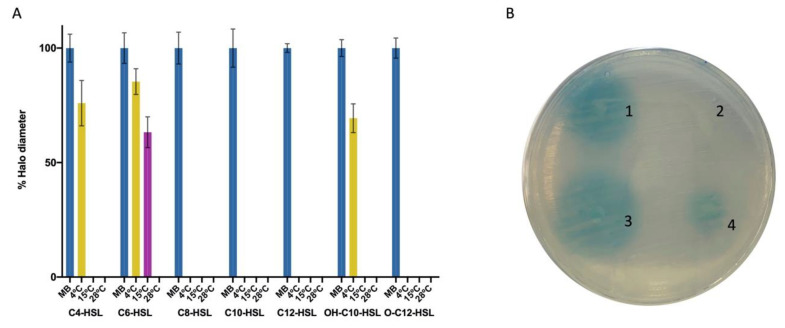
AHL-degradation activity of *Psychrobacter* sp. M9-54-1 explored using the agar plate diffusion assay method. (**A**) Degradation activity against synthetic AHLs, at different temperatures, expressed as percentage of halo diameter. Marine broth (MB) was used as negative control. (**B**) Detection of AHLs using biosensor strain *Agrobacterium tumefaciens* NTL4 (pZLR4): *V. owensii* VibC-Oc-106 crude extract (1), M9-54-1 degradation of *V. owensii* VibC-Oc-106 crude extract (2), *P. aeruginosa* PAO1 crude extract (3) and M9-54-1 degradation of a crude extract of *P. aeruginosa* PAO1 (4).

Strain M9-54-1, belonging to *Psychrobacter* sp., had previously been isolated from the microbiota of *Holothuria* spp. [38] and selected for its ability to degrade C6-homoserine lactone (HSL) and C10-HSL. The QS interference mechanism has been reported to involve enzymatic inactivation of AHLs but not the production of QSI compounds [24]. In the present study, in order to evaluate M9-54-1 AHL-degradation activity in more detail, a wide range of AHLs, including both synthetic and crude AHL extracts, was tested using agar well diffusion assays (Figure 1). Reactions with synthetic AHLs (C4-, C6, C8-, C10-, 3-OH-C10-, C12-, and 3-O-C12-HSL) were carried out at 4, 15, and 28 °C; the remaining AHL signal activity was measured as the diameter of colored halos developed by Agrobacterium tumefaciens biosensor strain NTL4 (pZLR4). Strain M9-54-1 showed activity against all AHLs at most temperatures, with or without substituted groups (Figure 1). Given that alkaline pH has been shown to drive AHL lactonolysis [31], pH was measured in all cultures to ensure that it was not the cause of AHL inactivation. Since all the AHLs analyzed were completely degraded at 28 °C, the QQ activity of *Psychrobacter* sp. M9-54-1 was tested at this temperature against AHL crude extracts from pathogenic *Vibrio* spp. and *Pseudomonas aeruginosa*. AHLs produced by aquaculture-related pathogens *V. owensii* VibC-Oc-106 (Figure 1), *V. mediterranei* VibC-Oc-097, and *V. coralliilyticus* VibC-Oc-193 (data not shown) were found to be completely degraded by M9-54-1. In the case of AHLs produced by the human pathogen *P. aeruginosa* PAO1, incubation with M9-54-1 resulted in extensive AHL degradation.

### 2.2. Whole-Genome Analysis of Psychrobacter sp. M9-54-1 Shows Potential for a New Species

With its partial 16S rRNA gene homology (99.21% identity) but only 43.7% genome sequence completeness, strain M9-54-1 had previously been identified as *Psychrobacter faecalis*. In this study, the genomic DNA of *Psychrobacter* sp. M9-54-1 was extracted and sequenced. Draft genome assembly led to 3.2 Kb in 69 contigs, with an average coverage of 230 X and G+C content of 43.4 mol%. The genome was deposited in the NCBI genome database under accession number JADGFW000000000. Having obtained the whole genome sequence, we were able to identify and analyze the complete 16S rRNA gene sequence of strain M9-54-1, which shares 99.78% identity with *P. pulmonis* CECT 5989^T^ and 99.66% identity with *P. faecalis* ISO-46^T^. The in silico DNA-DNA hybridization (DDH) and the average nucleotide identity (ANI) between strains M9-54-1 and *P. faecalis* SHUES1 (NZ_LXQA00000000.1), whose genome was available, were determined. The in silico DDH value was 60.4% and the ANI values based on ANIb and ANIm were 94.74 and 95.21%, respectively. Both DDH and ANI results were lower than the proposed cut-off values to describe a novel species (DDH, 70% and ANI, 95–96%), suggesting that strain M9-54-1 is a new species of the genus *Psychrobacter*. Nevertheless, no definitive conclusion can be drawn until the complete genome of *P. pulmonis* is published and compared with that of M9-54-1.

### 2.3. Psychrobacter sp. M9-54-1 AHL-Degrading Activity Is Cell-Associated and Not Due to Lactonolysis

**Figure 2:**
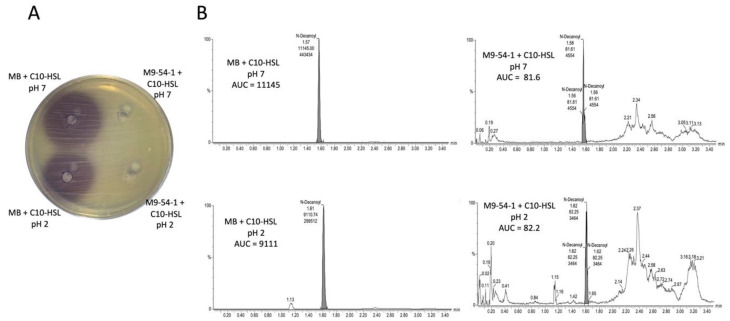
Detection of the remaining C10-HSL activity by agar well diffusion assay using *C. violaceum* VIR07 (**A**) and HPLC-MS (**B**) before and after *Psychrobacter* sp. M9-54-1 supernatant acidification to determine the quorum-quenching enzymatic mechanism present in this bacterium. Marine broth (MB) was used as negative control. HPLC values are referred to as area under the curve (AUC).

To determine whether the QQ activity of strain M9-54-1 is due to an AHL lactonase, an acidification assay was performed using C10-HSL, an AHL that was completely degraded by this strain. The concentration of AHLs was not restored in the acidified and filtered supernatant when they were tested by agar well diffusion assay (Figure 2A). To confirm this result, the AHL concentration was also analyzed by HPLC-MS (Figure 2B). The QQ activity of strain M9-54-1 was observed to be 100%, with no recovery in AHL concentration being detected following incubation under acidic conditions. These findings suggested that the mechanism by which autoinducers are degraded was not caused by an AHL lactonase.

The cellular localization of the enzyme was also evaluated by testing C10-HSL-degrading activity in supernatants and crude cellular extracts (CCEs) from M9-54-1 cultures using the agar well diffusion assay method. QQ activity was detected in CCEs, whilst no AHL degradation activity was detected in filtered supernatants, indicating that the enzyme is not secreted (data not shown).

### 2.4. Genome Analysis of Strain M9-54-1 Reveals that the AhaP Gene Is a Potential Acylase with AHL-Degrading Activity

To identify the gene responsible for QQ activity in *Psychrobacter* sp. strain M9-54-1, a BLASTp search was carried out against a database of predicted proteins from the genome of this strain, using a group of 28 well-known QQ enzymes as queries (Supplementary Table S1). Two proteins (Nos. 2034 and 322), presented in Table 1, were found to have an e-value of under 1 × 10^−100^ with different QQ enzymes, a threshold high enough to suggest that the hit could constitute a QQ enzyme.

The automatic annotation of the two proteins was then confirmed by RAST. Protein no. 2034 had been automatically annotated as acyl-homoserine lactone acylase PvdQ, while protein no. 322 was annotated as a hypothetical protein.

The corresponding genes 2034 (2.46 kb) and 322 (1.77 kb) were cloned into vector pGEX-4T-2, resulting in the production of pGEX-2034 and pGEX-322, respectively, and expressed in *E. coli* BL21 (DE3). Since only the expression of gene 2034 demonstrated an ability to degrade C10-HSL, as determined by the agar well diffusion assay method, gene 322 was discarded (data not shown). Protein No. 322 had homology with BpiB05, a QQ enzyme from an uncultured bacterium, and little is known about the key aminoacids for its activity, which may explain why no QQ activity was found in Protein No. 322. Thus, the protein encoded by gene 2034 was selected for further study and named acyl-homoserine lactone acylase from *Psychrobacter* sp. (AhaP).

**Figure 3:**
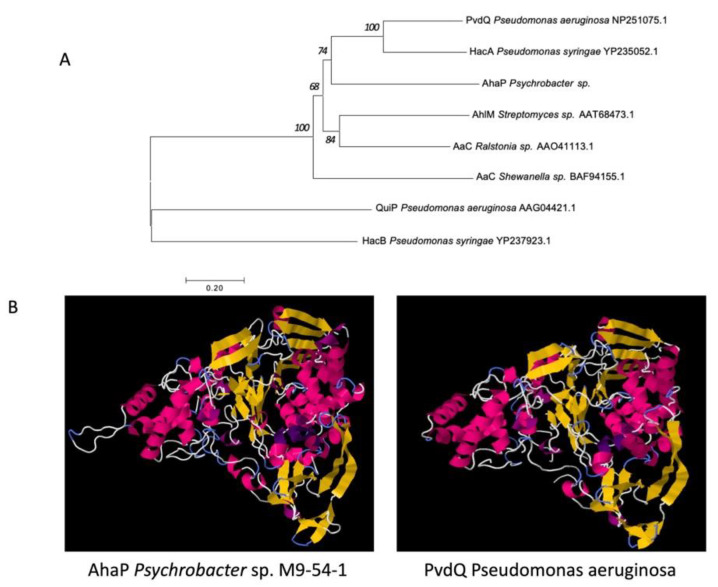
(**A**). Phylogenetic analysis of the acylase AhaP from *Pyschrobacter* sp. M9-54-1 and other known acylases using the neighbor-joining method with 1000 bootstrap replications. (**B**). Comparison of the predicted structures of the acylases AhaP from *Psychrobacter* sp. M9-54-1 and PvdQ from *Pseudomonas aeruginosa* PAO1 using the Phyre2 program.

The sequence of the predicted protein encoded by gene 2034 was compared with that of well-known QQ acylases with demonstrated AHL-degrading capacity using the neighbour-joining algorithm. The phylogenetic tree revealed that the enzyme AhaP from *Psychrobacter* sp. M9-54-1 shows high clustering similarity to other AHL acylases (Figure 3A). In fact, the predicted amino acid sequence of AhaP shared 36% identity with the amino acid sequence of PvdQ from *P. aeruginosa* PAO1 (NP_251075.1). As expected, the protein structure of AhaP, which was predicted using Phyre2 software, shared high similarity to that of the known acylase PvdQ from PAO1 (Figure 3B). The alignment between AhaP and PvdQ is shown in Supplementary Figure S1.

### 2.5. Purified AhaP Degrades AHLs with Different Substitutions and a Wide Range of Acyl Chain Lengths

**Figure 4:**
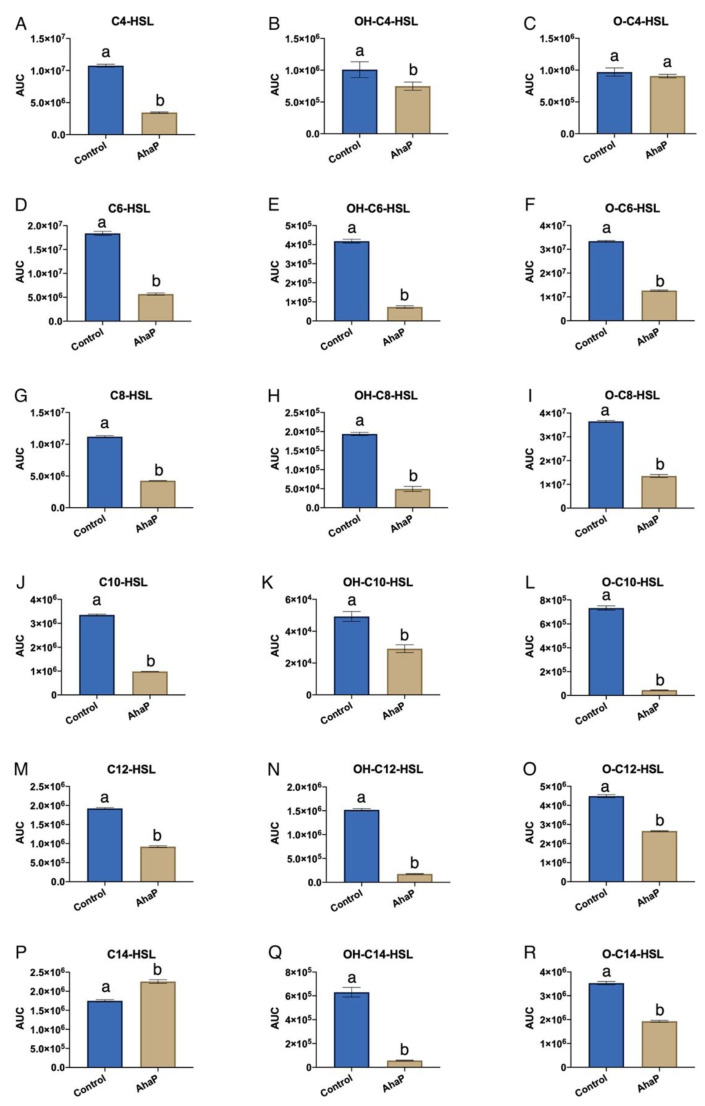
Quorum quenching activity of the purified enzyme AhaP against synthetic AHLs (C4-HSL (**A**), OH-C4-HSL (**B**), O-C4-HSL (**C**), C6-HSL (**D**), OH-C6-HSL (**E**), O-C6-HSL (**F**), C8-HSL (**G**), OH-C8-HSL (**H**), O-C8-HSL (**I**), C10-HSL (**J**), OH-C10-HSL (**K**), O-C10-HSL (**L**), C12-HSL (**M**), OH-C12-HSL (**N**), O-C12-HSL (**O**), C14-HSL (**P**), OH-C14-HSL (**Q**), O-C14-HSL (**R**). Autoinducer activity was detected with the biosensor strains *E. coli* (pSB536) for C4-HSL and its derivatives; *E. coli* (pSB401) for C6-, C8- and C10-HSL and their derivatives; and *E. coli* (pSB1142) for C12- and C14-HSL and their derivatives. Values are expressed as the area under the curve (AUC) of relative light units/OD_600_. Different letters above the bars indicate that the values are significantly different (*p* < 0.05).

To facilitate the purification of AhaP, the *ahaP* gene was cloned into vector pET-24b (+) with a C-terminal 6xHis tag and expressed in *E. coli* NiCo21. The use of this construct and host should improve the solubility and increase the yield. The purified protein, which integrity was analyzed by SDS-PAGE (data not shown), was used in a QQ assay with a wide range of synthetic AHLs (C4-, O-C4-, OH-C4-, C6-, O-C6-, OH-C6-, C8-, O-C8-, OH-C8-, C10-, O-C10-, OH-C10-, C12-, O-C12-, OH-C12-, C14-, O-C14-, and OH-C14-HSL). For each AHL, the remaining signal activity was detected by using the bioluminescent biosensor strains *E. coli* JM109 (pSB536) for C4-HSL and its derivatives *E. coli* JM109 (pSB401) for C6-, C8-, and C10-HSL and their derivatives, as well as E. *coli* JM109 (pSB1142) for C12- and C14-HSL and their derivatives. The results obtained demonstrated the presence of AhaP QQ activity against all the AHLs tested except for O-C4-, OH-C4-, and C14-HSL (Figure 4).

### 2.6. Heterologous Expression of ahaP in Pseudomonas aeruginosa Attenuates QS-Controlled Virulence Traits

**Figure 5:**
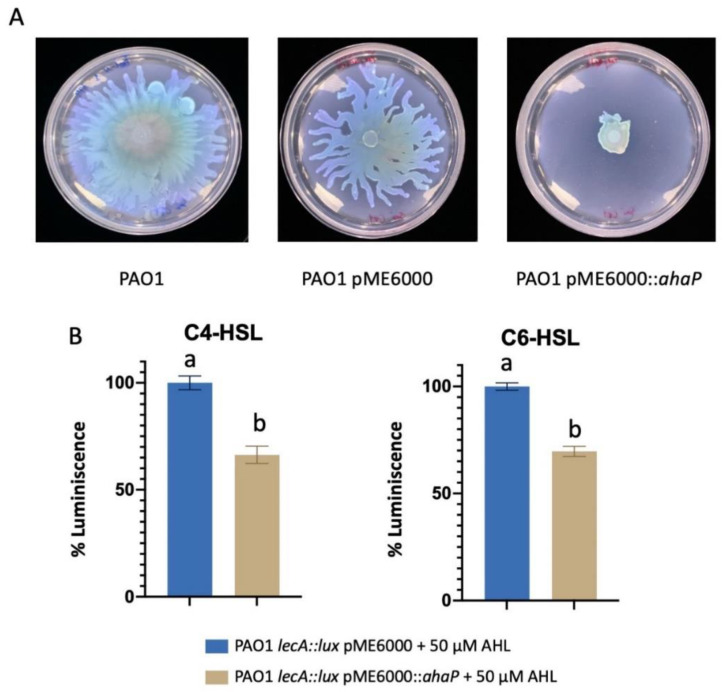
Evaluation of *ahaP* expression in *P. aeruginosa* PAO1. (**A**). Swarming motility assay of PAO1 wild type and strains containing the pME6000::*ahaP* construct and empty plasmid pME6000. (**B**). Detection of luminescence production in the biosensor strains PAO1 *lecA::lux* and PAO1 *lecA::lux* containing the pME6000::*ahaP* construct and empty plasmid pME6000 in the presence of C4- and C6-HSL. Different letters above the bars indicate that the values are significantly different (*p* < 0.05).

The *ahaP* gene was cloned into the broad host-range plasmid pME6000 and expressed in *Pseudomonas aeruginosa* PAO1 to evaluate its effect, if any, on phenotypes regulated by AHL-based QS systems. To this end, swarming motility of PAO1 overexpressing *ahaP* (pME6000::*ahaP*) was tested and compared to that of the same strain carrying the empty plasmid (pME6000) and the PAO1 wild type strain. As shown in Figure 5A, the expression of *ahaP* significantly reduced swarming motility in this opportunistic pathogen. Moreover, the pME6000::*ahaP* construct and empty plasmid were transferred to the biosensor strain PAO1 *lecA::lux* in which the expression of lectin gene *lecA* was evaluated. The strain’s bioluminescence output without plasmid pME6000 was also assessed as control. The luminescence produced by the reporter strain was significantly reduced (*p* < 0.05) when the incubation was carried out in the presence of exogenous C4- and C6-HSL which increase the biosensor response (Figure 5B). These results indicate that AhaP enzymatic activity reduces the expression of these two virulence factors in the human pathogen *P. aeruginosa* PAO1.

### 2.7. Psychrobacter sp. M9-54-1 Attenuates the Virulence of Vibrio Coralliilyticus VibC-Oc-193 Both In Vitro and In Vivo

**Figure 6:**
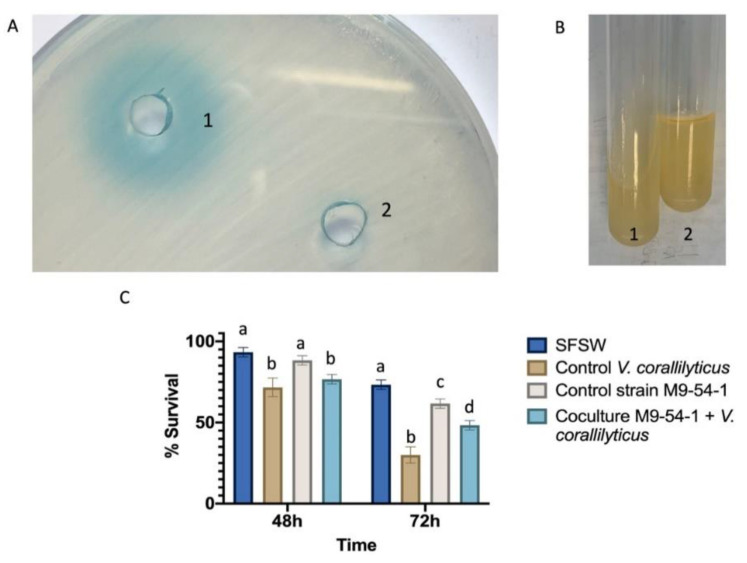
Production of AHLs (**A**) and gelatinase (**B**) by *V. coralliilyticus* VibC-Oc-193 (1) and the co-culture of *Psychrobacter* M9-54-1 and *V. coralliilyticus* VibC-Oc-193 (2). (**C**). Survival rate of *Artemia salina* nauplii after 48 h and 72 h of incubation with the different cultures. Different letters indicate significant differences (*p* < 0.05).

Given that *Psychrobacter* sp. M9-54-1 abolished AHL-mediated signal activity present in crude extracts from three *Vibrio* spp., the *V. coralliilyticus* strain VibC-Oc-193 was selected to test the effect of AHL degradation by M9-54-1 on virulence factors produced by this aquaculture pathogen. Under the conditions tested, the AHLs produced by *V. coralliilyticus* VibC-Oc-193 (Figure 6A) were degraded in the presence of the M9-54-1 strain. In the phenotypic analyses carried out, only the production of gelatinase in *V. coralliilyticus* VibC-Oc-193 was drastically reduced (Figure 6B).

To evaluate its potential use to control bacterial infections, we tested the QQ activity of M9-54-1 in a disease model of *Artemia salina* brine shrimp after 48 and 72 h infection with *V. coralliilyticus* VibC-Oc-193. Statistically significant disease attenuation was observed after 72 h of infection, resulting in an increase of 17.5% in the survival rate of *Artemia salina* incubated with M9-54-1 as compared to the infected *Artemia salina* without the *Psychrobacter* strain (Figure 6C).

To confirm that the reduction in virulence recorded was due to QS interference and not to a growth inhibitory effect on VibC-Oc-193, colony forming units from M9-54-1 were assessed. No differences in VibC-Oc-193 viability were observed from either culture (data not shown), suggesting that the decrease in virulence observed is likely associated with AHL degradation by M9-54-1.

## 3. Discussion

During the last decade, antimicrobial resistance (AMR) has become a serious global threat to human and animal health. International organizations such as FAO and WHO are working closely on implementing a global plan to minimize the threat of AMR which includes the development of novel strategies to fight bacterial infection diseases (www.fao.org). One novel and promising approach in which the research community is interested is interference with quorum sensing which controls virulence gene expression in numerous pathogens. The majority of studies have focused on enzymatic AHL degradation, with only a few devoted to non-enzymatic inhibitory mechanisms [14].

The marine environment has proven to be an excellent source of novel enzymes and compounds that interfere with QS systems [11,40,41]. In an attempt to discover novel QS-inhibiting compounds, we previously explored the marine symbiotic bacteria of invertebrates from which we selected AHL-degrading [24] and QSI-producing bacteria [39]. Thus, strain M9-54-1, with its high levels of AHL-degrading activity, was isolated from holothurians and selected for further study. In the present study, a cold-adapted QQ enzyme named AhaP, which was identified and purified from strain M9-54-1, demonstrated its ability to degrade with high levels of activity substituted and unsubstituted AHLs.

The strain M9-54-1 has been identified as belonging to the genus *Psychrobacter* based on the 16S rRNA gene sequence and whole genome sequencing. Its full-length 16S rRNA gene sequence similarity to *P. faecalis* and *P. pulmonis* was over 99.5%, indicating that this strain could belong to either of these species. However, a whole genome comparison between M9-54-1 and *P. faecalis* based on digital DNA-DNA hybridization and ANI values, suggests that M9-54-1 could be a new species of the genus *Psychrobacter* [42]. Since the whole genome sequence of *P. pulmonis* was not available, a whole-genome comparison with this species could not be performed. Interestingly, despite high similarities amongst their 16S rRNA gene sequences, these species differ in terms of phenotypic characteristics such as carbohydrate use [43].

Most bacteria of the genus *Psychrobacter* are psychrotolerant, with a growth temperature ranging from −10 to 38 °C, and halotolerant, showing growth in the presence of up to 6.5% (*w/v*) NaCl. Many of these bacteria are isolated from cold and high-salt environments such as Antarctic and seawater [44]. Psychrophile and psychrotolerant bacteria have become important vehicles for isolating novel high-activity enzymes at low temperatures, making them excellent energy-saving biocatalysts with a reduced environmental impact [45]. In addition, these enzymes are more thermostable than those originating from plants and animals [46].

Cold-adapted enzymes are considered to have potential biotechnological and industrial applications that require activity at mild temperatures and rapid heat inactivation rates, such as that found in molecular biology, medical research, food/feed processing, pharmaceuticals, detergents and cosmetics [45,47,48,49]. With regard to *Psychrobacter,* cold-adapted enzymes, such as ribonucleases, proteases, esterases and lipases, have been found in some species of this genus [45,47,50,51,52]. Nevertheless, the production of compounds that interfere with quorum sensing (QS) in *Psychrobacter* strains has been the subject of a limited number of studies. For example, a marine sponge *Psychrobacter* sp. strain was found to degrade 3-O-C8-HSL and to be able to reduce swarming motility in *Pseudomonas aeruginosa* [53]. Another strain of *Psychrobacte*r sp. isolated from a marine sponge has been reported to produce cyclic dipeptides that act as QS inhibitors via a non-enzymatic mechanism [54]. In our study, the experimental evidence presented, together with a phylogenetic analysis of the AhaP protein, suggest that the QQ enzyme produced by strain M9-54-1 belongs to a family of AHL acylases, showing high sequence and structural similarity to PvdQ from *P. aeruginosa* [55]. These findings reinforce the notion that the QS interference mechanism of strain M9-54-1 is not caused by the production of compounds that block the interaction of AHLs with their cognate signal receptors.

*Psychrobacter* sp. strain M9-54-1 has shown high levels of activity against a broad range of synthetic AHLs as well as those produced by bacterial pathogens. Thus, the potential use of AhaP enzyme to combat bacterial infections was evaluated by expressing it in the human pathogen *P. aeruginosa* PAO1. A recurrent problem associated with infections caused by *P. aeruginosa* is the multiple antibiotic resistance displayed by clinical isolates of this bacterium [56,57], which greatly limits the treatment choices available. In this study, by using the strain *P. aeruginosa* PAO1 and the *P. aeruginosa* bioreporter PAO1 *lecA::lux*, we demonstrated that *ahaP* gene expression interferes with the production of the carbohydrate-binding protein LecA which acts as an adhesin and a cytotoxin [58]. The reduction in *lecA* gene expression when PAO1 cultures were spiked with AHLs, as well as swarming motility inhibition, a well-studied QS-regulated phenotype in *P. aeruginosa* [59], suggest the potential use of AhaP as an anti-virulence treatment technique.

Strain M9-54-1, which is a marine bacterium, was also tested as a potential biocontrol tool to combat infections in aquaculture. The QS system is known to be involved in virulence control mechanisms of *Vibrio* spp. whose interference has proved to be a promising strategy to control vibriosis, a bacterial disease which has a considerable impact on aquaculture [60,61]. Prior to the in vivo experiments, co-cultures between strain M9-54-1 and *V. coralliilyticus* VibC-Oc-193 were conducted, leading to a drastic reduction in AHLs and gelatinase production in the pathogen. Other virulence factors such as enzymatic activities produced by the pathogen were also tested but no significant differences were observed. The 100:1 QQ bacterium to pathogen ratio used in the co-culture experiments in order to obtain successful results was similar to that used in previous studies [24,37,62]. Thus, in vivo experiments were conducted under conditions similar to those used in the co-culture assays in order to test *V. coralliilyticus* VibC-Oc-193 virulence using *Artemia salina* nauplii in an animal model which have been used in previous studies [10,24,63,64]. Strain M9-54-1 significantly reduced the mortality of *Artemia salina* nauplii after 72 h of infection with the pathogen, making it an excellent potential biocontrol agent in aquaculture.

Numerous studies have reported the effect of AHL-degrading bacteria, as well as heterologous QQ gene expression, on the reduction in the production of pathogen virulence factors in humans, aquaculture and agriculture [11,30,65,66]. Nevertheless, little is known about cold-adapted AHL-degrading enzymes, referred to as lactonases. For example, several cold-adapted lactonase-producing *Planococcus* strains isolated from Antarctica have been identified. These include the cold-adapted AHL lactonase AidP which was purified and demonstrated to attenuate the pathogenicity of *Pectobacterium carotovorum* [67]. Another cold-adapted AHL lactonase, named Aii810, derived from the high QQ activity Mao-tofu metagenome at low temperatures, was found to decrease virulence factors and biofilm in *P. aeruginosa* PAO1 [68]. In this study, a cold-adapted acylase-type QQ enzyme produced by a *Psychrobacter* strain has been characterized for the first time. Cold-adapted enzymes used as biotechnological tools, which are cost-effective and catalyze reactions at low temperatures, offer advantages over their mesophilic homologues, by reducing bacterial contamination and slowing down undesirable chemical reactions. Moreover, many of these thermolabile enzymes can be inactivated by moderate heat instead of chemical-based treatment [47].

In conclusion, in this study, a cold-adapted acylase enzyme named AhaP from M9-54-1, a marine strain of the genus *Psychrobacter,* has been identified for the first time. After purification, this enzyme showed high levels of activity against a broad range of AHLs and significantly reduced the production of virulence factors in *P. aeruginosa*. In vivo assays also demonstrated that M9-54-1 reduces the virulence of *V. coralliilyticus* VibC-Oc-193, increasing the survival rate of *A. salina* infected with this pathogen by 17.5%. This result reinforces the potential of using strain M9-54-1 as a biocontrol agent to combat vibriosis in aquaculture.

## 4. Materials and Methods

### 4.1. Bacterial Strains, Media and Culture Conditions

The strain M9-54-1 was routinely grown at 28 °C in marine broth (MB, BD Difco^®^, Franklin Lakes, NJ, USA). The biosensor strain *Agrobacterium tumefaciens* NTL4 (pZLR4) [69] was cultured at 28 °C in Luria-Bertani (LB) medium supplemented with 2.5 mmol L^−1^ CaCl_2_ and 2.5 mmol L^−1^ MgSO_4_ (LB/MC) or AB medium (3 g K_2_HPO_4_, 1 g Na_2_H_2_PO_4_, 1 g NH_4_Cl, 0.3 g MgSO_4_·7H_2_O, 0.15 g KCl, 0.01 g CaCl_2_, 0.0025 g FeSO_4_·7H_2_O and 5 g glucose per liter) containing 50 µg gentamycin mL^−1^. *Chromobacterium violaceum* CV026 [70] and *C. violaceum* VIR07 [71] were grown at 28 °C in LB medium supplemented with kanamycin (50 µg mL^−1^). The biosensor strains *Escherichia coli* JM109 (pSB536), *E. coli* JM109 (pSB401) and *E. coli* JM109 (pSB1142) [72] were grown in the presence of ampicillin (100 µg mL^−1^) or tetracycline (20 or 10 µg mL^−1^) as required [72].

The AHL-producing bacteria used for in vitro and in vivo tests were *Pseudomonas aeruginosa* PAO1 [73] and the aquaculture-related pathogenic species *Vibrio mediterranei* VibC-Oc-097, *V. owensii* VibC-Oc-106 and *V. coralliilyticus* VibC-Oc-193 [10,74]. *P. aeruginosa* PAO1 was grown in LB at 37 °C and *Vibrio* species were grown in MB at 28 °C.

### 4.2. QQ Activity upon Synthetic AHLs and Crude AHL Extracts from Pathogenic Bacteria

The capacity of M9-54-1 to degrade a wide range of synthetic AHLs as well as crude AHL extracts from pathogenic bacteria was studied using agar well diffusion assays [34,37]. The synthetic AHLs used were C4-HSL (*N*-butyryl-DL-homoserine lactone), C6-HSL (*N*-hexanoyl-DL-homoserine lactone), C8-HSL (*N*-octanoyl-DL-homoserine lactone), C10-HSL (*N*-decanoyl-DL-homoserine lactone), 3-OH-C10-HSL (*N*-3-hydroxydecanoyl-DL-homoserine lactone), C12-HSL (*N*-dodecanoyl-DL-homoserine-lactone) and 3-O-C12-HSL (*N*-3-oxo-dodecanoyl-DL-homoserinelactone) (Sigma^®^, St. Louis, Missouri, USA). These AHLs were added to 500 µL of a 24 h culture of strain M9-54-1, at a final concentration of 10 µM. After 24 h of incubation at different temperatures (4, 15 and 28 °C), the remaining signals were detected by loading 100 µL of cell-free supernatant in agar plate wells seeded with the AHL biosensor strains. *A. tumefaciens* NTL4 (pZLR4) and AB agar plates supplemented with 80 µg mL^−1^ of 5-bromo-4-chloro-3-indolyl-ß-D-galactopyranoside (X-Gal) were used to detect C12-HSL and 3-O-C12-HSL activity; *C. violaceum* CV026 and VIR07 strains were used to detect C4-HSL, C6-HSL, C8-HSL, C10-HSL and 3-OH-C10-HSL signals.

To prepare AHL extracts from pathogenic vibrios or *P. aeruginosa* PAO1, a previously described methodology was followed [75,76]. Briefly, 500 µL of 24 h cultures of each AHL-producing pathogenic bacterium were extracted twice with one volume of dichloromethane or acidified ethyl acetate. The extract was dried and resuspended in 20 or 50 µL of methanol 70% (*v*/*v*). Crude extracts were then stored at −20 °C prior to testing.

To evaluate the ability of M9-54-1 to degrade AHL signals present in extracts from *Vibrio* spp. and *P. aeruginosa* PAO1, 5 µL of the AHL crude extracts were added to 1 mL of an overnight culture of M9-54-1. After 24 h of incubation, the remaining AHLs were extracted twice with one volume of dichloromethane, dried and resuspended in 20 µL of methanol 70% (*v*/*v*). These extracts were spotted onto Whatman^®^ (GE Healthcare, Chicago, IL, USA) sterile disks placed in AB agar plates supplemented with X-Gal in which an A. *tumefaciens* NTL4 (pZLR4) lawn had been previously seeded. After 24 h of incubation, the appearance of blue halos was visually inspected.

### 4.3. Identification of the Type and Location of QQ Activity

An acidification assay [31,77] was carried out to determine whether the QQ activity of M9-54-1 was due to a lactonase mechanism. Briefly, C10-HSL was added to 500 µL of a 24 h culture of M9-54-1 at a final concentration of 10 µM. After 24 h of incubation, the mixtures were centrifuged and the pH of the supernatants was adjusted to 2.0 with HCl 1N. Acidified supernatants were then incubated at 28 °C for 24 h and the presence of AHL activity in dichloromethane extracts from the reaction mixtures was assessed using *C. violaceum* CV026.

To identify the cellular localization of AHL QQ activity present in M9-54-1, supernatants and crude cellular extract (CCE) fractions from 24 h cultures of M9-54-1 were obtained and assayed for QQ activity as described previously [78]. The CCEs were obtained by centrifugation, resuspension of the pellet in PBS buffer (pH 6.5) and sonication of the suspension for 5 min. Afterwards, the CCEs and culture supernatants were filtered through a 0.22-µm pore membrane filter. A QQ assay was carried out with these two fractions as described in Section 2.2.

### 4.4. Detection of AHL Degradation Activity by HPLC-MS

The AHL QQ activity of *Psychrobacter* strain M9-54-1 against C10-HSL was studied using HPLC-MS according to a previously described methodology [36]. The QQ reaction was performed as described in Section 2.2 and the remaining signal was extracted twice in 500 µL of dichloromethane, with or without prior acidification as described in Section 2.3. Dried extracts were then resuspended in 500 µL of acetonitrile and analyzed by HPLC-MS.

### 4.5. DNA Extraction and Whole-Genome Sequencing Analysis

The genomic DNA of strain M9-54-1, which was extracted using a methodology described elsewhere [79], was sequenced at the STAB Vida facility (Caparica, Portugal) using Illumina Mi-Seq technology. The low-quality bases and adaptors were trimmed using the bbduk program (https://jgi.doe.gov/data-and-tools/bbtools/) and the reads were assembled using Spades v3.11. Digital DNA-DNA hybridization was calculated using the DSMZ web server (http://ggdc.dsmz.de) [80], while ANIb and ANIm calculations were performed on the JSpeciesWS website [81].

To search for potential QQ enzymes, the whole genome of strain M9-54-1 was annotated using RAST [82,83] and genes encoding potential QQ enzymes were selected using BLASTp [84] and a database of 28 QQ enzymes as queries (Supplementary Table S1).

### 4.6. Cloning of Genes Encoding Quorum Quenching Activity

Potential QQ genes identified in the genome of *Psychrobacter* sp. strain M9-54-1 were amplified using Q5 HF DNA polymerase (New England BioLabs^®^, Hitchin, UK) with primers including *Bam*H1 and *Sal*1 sites: 2034-F/R 5′-GGATCCATGAGCATTAATGTGCTTAATC-3′/5′-GTCGACCTATTC TCTCAATTTGATCACT-3′, and 322-F/R 5′-GGATCCATGACAAAAATAATCGACTA-3′/5′-GTCGACTTAACGATTAAGTAAATCTGTG-3′. PCR products were purified and cloned into pGEM-T Easy Vector (Promega^®^, Fitchburg, WI, USA) according to the manufacturer’s recommendations. The vector was transferred to *Escherichia coli* DH5α and extracted using the New England Biolabs^®^ Monarch Miniprep Kit. The pGEM-T vector carrying the insert, as well as the pGEX-4T-2 plasmid, were digested using the above-mentioned restriction enzymes, and the inserts were ligated in the expression vector pGEX-4T-2. This expression vector was transformed in *E. coli* BL21 (DE3) and clone activity was tested using the agar well diffusion assay as previously mentioned (Table 2).

The amino acid sequence of the putative AHL-acylase encoded by gene 2034 of M9-54-1 and sequences from other known acylases were used to build a phylogenetic tree using MEGA7 software [85]. Phyre2 was used to predict the structure of QQ enzymes [86].

### 4.7. Expression and Purification of Recombinant ahaP

The *ahaP* gene was amplified with the primers including *Bam*H1 and *Xho1* sites: *ahaP*-F/R 5′ TATGGATCCGTGTTCGGATAACGACAATTTTA3′/5′TATCTCGAGTTCTCTCAATTTGATCACTTCA3′. The purified insert was digested with the indicated restriction enzymes, ligated in pET-24b(+) and transformed into *E. coli* NiCo21 (DE3), which is specially designed for the expression of histidine-tagged recombinant proteins (Table 2).

500 mL of LB were inoculated with 10 mL of a 24 h culture of *E. coli* NiCo21 (DE3) (pET-24b(+)::*ahaP*) and incubated at 37 °C. Once OD_600_ reached 0.6, the culture was induced with 1 mM IPTG and further incubated for 5 h at 37 °C. The pellet obtained by centrifugation was stored at -80 °C prior to AhaP purification using the Ni-NTA Fast Start Kit (Qiagen^®^). The integrity of the purified protein was analyzed by SDS-PAGE and stored at −20 °C until use.

### 4.8. Detection of AHL Degradation Activity of AhaP

50 µL of purified AhaP enzyme at concentration 0.5 mg/mL were added to 450 µL of PBS supplemented with 5 µM of each of the following AHLs: C4-HSL (*N*-butyryl-L-homoserine lactone), O-C4-HSL (*N*-3-oxo-butyryl-L-homoserine lactone), OH-C4-HSL (*N*-hydroxybutyryl-L-homoserine lactone), C6-HSL (*N*-hexanoyl-L-homoserine lactone), O-C6-HSL (*N*-3-oxo-hexanoyl-L-homoserine lactone), OH-C6-HSL (*N*-hydroxy hexanoyl-L-homoserine lactone), C8-HSL (*N*-octanoyl-L-homoserine lactone), O-C8-HSL (*N*-3-oxo-octanoyl-L-homoserine lactone), OH-C8-HSL (*N*-hydroxyoctanoyl-L-homoserine lactone), C10-HSL (*N*-decanoyl-L-homoserine lactone), O-C10-HSL (*N*-3-oxo-decanoyl-L-homoserine lactone), 3-OH-C10-HSL (*N*-3-hydroxydecanoyl-L-homoserine lactone), C12-HSL (*N*-dodecanoyl-L-homoserine-lactone), 3-O-C12-HSL (*N*-3-oxo-dodecanoyl-L-homoserine lactone), 3-OH-C12-HSL (*N*-3-hydroxydodecanoyl-L-homoserine lactone), C14-HSL (*N*-tetradecanoyl-L-homoserine-lactone), 3-O-C14-HSL (*N*-3-oxo-tetradecanoyl-L-homoserine lactone) and 3-OH-C14-HSL (*N*-3-hydroxytetradecanoyl-L-homoserine lactone). AHLs were synthesized at the University of Nottingham. PBS without added enzyme was used as control.

Reactions were incubated overnight at 37 °C and 20 µL were mixed with 80 µL of fresh LB and loaded into 96-well plates. 100 µL of a subculture (OD_600_ = 0.01) of the biosensors *E. coli* JM109 pSB536 was used for detection of C4-, O-C4- and OH-C4-HSL; *E. coli* JM109 pSB401 for C6-, O-C6-, OH-C6-, C8-, O-C8-, and OH-C8-HSL; and *E. coli* JM109 pSB1142 for long chain AHLs. Absorbance at 600nm and luminescence of each well were recorded for 24 h and the AUC of relative luminescence ratios (light units/OD_600_) was calculated. The assay was carried out in triplicate.

### 4.9. AHL-Acylase Gene Expression in Pseudomonas Aeruginosa PAO1

The AHL-acylase gene *ahaP* of strain M9-54-1 was ligated into the broad-host-range plasmid pME6000. The DNA construct and the empty plasmid were then transformed in wild type *P. aeruginosa* PAO1 and in *P. aeruginosa* PAO1 *lecA::lux*, a reporter strain regulated by QS [58] (Table 2).

Swarming motility assays were performed using freshly prepared agar plates of swarming media (Nutrient Broth No.2 0.8% *w*/*v*, glucose 0.5% *w*/*v*, agar 0.5% *w*/*v*). The wild type strain PAO1 without plasmid pME6000 was used as control. A subculture with an OD_600_ = 0.5 of PAO1^WT^, PAO1 (pME6000), and PAO1 (pME6000::*ahaP*) was prepared and 3 µL of the subcultures were inoculated in the center of the plates and incubated at 37 °C overnight to evaluate swarming motility.

In the case of *P. aeruginosa* PAO1 *lecA::lux*, a subculture in LB with OD_600_ = 0.02 of *P. aeruginosa* PAO1 *lecA::lux, P. aeruginosa* PAO1 *lecA::lux* (pME6000) and *P. aeruginosa* PAO1 *lecA::lux* (pME6000::*ahaP*) was prepared and supplemented with C4 or C6-HSL at different concentrations. They were placed in 96-well plates with black walls, and OD_600_ absorbance and luminescence were recorded every 30 min. After 24 h of incubation, the AUC of the RLU/OD_600_ ratio was calculated.

### 4.10. In Vitro Co-Culture Assays

Co-culture assays were carried out with *Psychrobacter* strain M9-54-1 and pathogenic *Vibrio* spp. [24]. Briefly, 50 µL of a 24 h culture of the pathogen were added to 5 mL of a 24 h culture of *Psychrobacter* M9-54-1. After 24 h of co-incubation, AHLs produced in the co-cultures were detected using the well-diffusion assay method as described above. To evaluate the interference of *Psychrobacter* M9-54-1 with virulence factor expression in the pathogenic strain, co-cultures were tested for a diverse range of enzymatic activities by spotting 10 µL of the co-cultures on different media. Proteolytic and haemolytic activity was determined in casein medium and in blood agar medium, respectively [90]. Acid phosphatase activity was determined in PVK medium [91], whilst alkaline phosphatase was determined with MA or LB supplemented with phenolphthalein phosphate 0.01% (*w*/*v*) [92]. Amylase activity was determined in MA supplemented with starch 1% (*w*/*v*) [93]. Gelatinase activity was detected using a gelatin medium (BD Difco^®^, Franklin Lakes, NJ, USA). The hydrolysis of esculin was tested in MA supplemented with 0.5% (*w*/*v*) esculin and 0.05% (*w*/*v*) ferric chloride added [94].

### 4.11. In Vivo Assays against Vibrio Coralliilyticus

The virulence of *V. coralliilyticus* VibC-Oc-193 in the absence and presence of strain M9-54-1 was tested in vivo in *Artemia salina* (brine shrimp) nauplii according to a protocol described elsewhere [10,24]. Briefly, the hatching cysts of *A. salina* (JBL Artemio Pur, JBL GmbH & Co. KG, Neuhofen, Germany) were obtained according to the manufacturer’s instructions using sterile-filtered and autoclaved seawater (SFSW) (salinity 36 g L^−1^, 20 °C, pH 7.3), and groups of 20 nauplii were transferred to petri dishes containing 20 mL of SFSW. Then, co-cultures were made with 5 mL of 24-h culture of M9-54-1 to which 50 µL of a 24 h culture of *V. coralliilyticus* were added. As controls, monocultures of strain M9-54-1, *V. coralliilyticus* and the same volume of SFSW were used. For each condition, the bacterial cells were washed with SFSW and added to the shrimp larvae in a final concentration of 10^6^ CFU mL^−1^, with cell number determined by the plate counting method, and were then incubated at 25 °C for 3 days. Each condition was done in triplicate. The survival of the shrimps, detected by loss of motility, was scored daily after the addition of bacteria. All results were analyzed using the ANOVA test (*p* < 0.05) followed by Tukey´s *t*-test using R software (3.6.2) (The R Foundation, Vienna, Austria).

## Figures and Tables

**Table 1:** Homologues to quorum quenching putative proteins identified in the genome of *Psychrobacter* sp. M9-54-1.

Homologues	Identity (%)	Positives (%)	e-Value
**Protein No. 2034**			
AaC *Ralstonia* sp. XJ12B (AAO41113.1)	37	54	3.00 × 10^−171^
AaC *Shewanella* sp. MIB015 (BAF94155.1)	32	47	1.00 × 10^−112^
AhlM *Streptomyces* sp. M664 (AAT68473.1)	35	52	1.00 × 10^−131^
HacA *Pseudomonas psyringae* pv. *syringae* B728a (YP_235052.1)	35	53	4.00 × 10^−152^
PvdQ *Pseudomonas aeruginosa* PAO1 NP_251075.1	36	53	1.00 × 10^−162^
**Protein No. 322**			
Uncultured bacterium BpiB05 Bio8 (ABU51109)	40	59	3.00 × 10^−159^

**Table 2:** Bacterial strains and plasmids used in this study.

Strain or Plasmid	Genotype or Description	Source or Reference
**Strains**		
*Escherichia coli*		
DH5α	*rel*A1, *lacZ*∆M15, *sup*E44, *thi-1*	[87]
XL1-Blue	*sup*E44 *hsd*R17 *rec*A1 endA1 ggyrA46 *thi* relA1 *lac^-^* F’ (*pro*AB^+^ *lac*I^q^ *lac*ZDM15 Tn*10(*tet^r^*))*	[88]
BL21(DE3) pLysS	Cm^R^, *dcm omp*T *hsdS* gal λ(DE3)	Promega^®^
NiCo21 (DE3)	*can::CBD fhuA2 [lon] ompT gal (λ DE3) [dcm] arnA::CBD slyD::CBD glmS6Ala ΔhsdS λ DE3 = λ sBamHIo ΔEcoRI-B int::(lacI::PlacUV5::T7 gene1) i21 Δnin5*	New England Biolabs^®^
*Pseudomonas aeruginosa*		
PAO1	Wild type	[73]
PAO1 *lecA::lux*	*lecA*::*luxCDABE* genomic reporter fusion in PAO1	[58]
**Plasmids**		
pGEM-T	High-copy-number cloning vector, Ap^R^, *bla*, *lacZ*	Promega^®^
pG2034	pGEM-T with a 2.46-kb PCR *Bam*H1-*Sal*1 fragment amplified from strain M9-54-1 genome containing gene-encoding protein No.2034, Ap^R^	This work
pG322	pGEM-T with a 1.77-kb PCR *Bam*H1-*Sal*1 fragment amplified from strain M9-4-1 genome containing gene-encoding protein No.233, Ap^R^	This work
pGEX-4T-2	Glutathione-S-transferase fusion vector, Ap^R^	GE-Healthcare^®^
pGEX-2034	pGEM-4T-2 with a 2.46-kb *Bam*HI-*Sal*I fragment containing gene-encoding protein No.2034, Ap^R^	This work
pGEX-233	pGEM-4T-2 with 1.77-kb *Bam*HI-*Sal*I fragment containing gene-encoding protein No.233, Ap^R^	This work
pET-24b (+)	Cloning vector carrying a C-terminal His-Tag^®^ sequence, Ap^R^, Km^R^	Novagen^®^
pET-24b(+)::*ahaP*	pET24b (+) containing *ahaP*	This work
pME6000	Broad-host-range expression vector, Tc^R^	[89]
pME6000::*ahaP*	pME6000 containing *ahaP*	This study

## Data Availability

The data presented in this study are fully available in the main text and Supplementary Materials of this article.

## Supplementary Materials

The following material is available online at https://www.mdpi.com/1660-3397/19/1/16/s1, Table S1: Quorum quenching enzymes used in this study as queries in the BLASTp search for the identification of the QQ enzyme in *Psychrobacter* sp. M9-54-1. Supplementary Figure S1. Alignment of the protein sequences of AhaP from *Psychrobacter* sp. M9-54-1 and PvdQ (NP_251075.1) from *Pseudomonas aeruginosa* PAO1.
