# Supplementary material for: AhaP, A Quorum Quenching Acylase from Psychrobacter sp. M9-54-1 That Attenuates Pseudomonas aeruginosa and Vibrio coralliilyticus Virulence
Source: Mar Drugs. 2021 Jan 1;19(1):16. doi: 10.3390/md19010016 (PMC7823738; doi:10.3390/md19010016)
Supplement: Supplementary file 1 [file marinedrugs-19-00016-s001.pdf]

Supplementary Materials:

AhaP, a quorum quenching acylase from Psychrobacter sp. M9-54-1 that attenuates *Pseudomonas aeruginosa* and *Vibrio coralliilyticus* virulence

José Carlos Reina[1], Manuel Romero[2], Rafael Salto[3], Miguel Cámara[2], Inmaculada Llamas[1,4*]

[1]Department of Microbiology, Faculty of Pharmacy, University of Granada, Campus Universitario Cartuja s/n, 18071 Granada, Spain.

[2]National Biofilms Innovation Centre, Biodiscovery Institute and School of Life Sciences, University of Nottingham, Nottingham, NG7 2RD, UK.

[3]Department of Biochemistry, Faculty of Pharmacy, University of Granada, Campus Universitario Cartuja s/n, 18071 Granada, Spain.

[4]Institute of Biotechnology, Biomedical Research Center (CIBM), University of Granada, 18100 Granada, Spain.

*Correspondence: illamas@ugr.es

Supplementary Table S1. Quorum quenching enzymes used in this study as queries in the BLASTp search for the identification of the QQ enzyme in *Psychrobacter* sp. M9-54-1.

Enzyme	Accession number	Species	Enzyme type	Reference
**AiiA**	AAM61772.1	*Bacillus* spp.	Lactonase (Metallo-beta-lactamases superfamily)	[1]
**AaC *Shewanella* sp.**	BAF94155.1	*Shewanella* sp. MIB015	Acylase (Ntn-hydrolase)	[2]
**AaC *Ralstonia* sp.**	AAO41113.1	*Ralstonia solanacearum* GMI1000	Acylase (Ntn-hydrolase)	[3]
**AhlD**	AAP57766.1	*Arhtobacter* sp. IBN110	Lactonase (Metallo-beta-lactamases superfamily)	[4]
**AhlK**	AAO47340.1	*Klebsiella pneumoniae* KCTC2241	Lactonase (Metallo-beta-lactamases superfamily)	[4]
**AhlM**	AAT68473.1	*Streptomyces* sp. M664	Acylase (Ntn-hydrolase)	[5]
**AhlS**	BAK54003.1	*Solibacillus silvestris* StLB046	Lactonase (Metallo-beta-lactamases superfamily)	[6]
**AidC**	BAP32158.1	*Chryseobacterium* sp. StRB126	Lactonase (Metallo-beta-lactamases superfamily)	[7]
**AiiM**	BAJ75775.1	*Microbacterium testaceum* StLB037	Lactonase (Metallo-beta-lactamases superfamily)	[7]
**AiiO**	ADI80348.1	*Ochrobactrum* sp. A44	Acylase (alfa/beta hydrolase)	[8]
**AttM**	AAD43990.1	*Agrobacterium tumefaciens* C58, M103	Lactonase (Metallo-beta-lactamases superfamily)	[9]
**Bpib01**	ABU51804.1	Metagenome	Hypothetical	[10]
**Bpib04**	ABU51107.1	Metagenome	Lactonase (Glycosyl hydrolase)	[10]
**Bpib05**	ABU51109.1	Metagenome	Hypothetical	[11]
**Bpib07**	ABU51111.1	Metagenome	Lactonase (Dienolactone hydrolase)	[10]
**HacA**	YP_235052.1	*Pseudomonas syringae* B728a	Acylase (Ntn-hydrolase)	[12]
**HacB**	YP237923.1	*Pseudomonas syringae* B728a	Acylase (Ntn-hydrolase)	[12]
**MCP**	AAS06218.1	*M. avium* subsp. *paratuberculosis* K-10	Phosphotriesterase-like lactonase (PLL)	[13]
**PON**	EAP_90803.1	*Oceanicaulis alexandrii* HTCC2633	Lactonase (Paraoxonase)	[14]
**PpH**	NP_214744.1	*Mycobacterium tuberculosis*	Phosphotriesterase-like lactonase (PLL)	[15]
**PvdQ**	NP_251075.1	*Pseudomonas aeruginosa* PAO1	Acylase (Ntn-hydrolase)	[16]
**QlcA**	ABV58973.1	Sin identificar *(metagenoma)*	Lactonase (Metallo-beta-lactamases superfamily)	[17]
**QsdA**	AAT06802.1	*Rhodococcus erythropolis* W2	Phosphotriesterase-like lactonase (PLL)	[15]
**QsdB**	AFX82613.1	Sin identificar *(metagenoma)*	Acilase (Amidase signature family)	[18]
**QsdH**	AFV15299.1	*Pseudoalteromonas byunsanensis*	Lactonase (GDSL-hidrolases family)	[19]
**QuiP**	AAG04421.1	*Pseudomonas aeruginosa* PAO1	Acylase (Ntn-hydrolase)	[20]
**Sislac**	Pdb|4G2D|A	*Sulfolobus islandicus*	No identified	[21]
**SsoPox**	Q97VT7.1	*Sulfolobus solfataricus* P2	No identified	[22]

Figure S1. Alignment of the protein sequences of AhaP from *Psychrobacter* sp. M9-54-1 and PvdQ (NP_251075.1) from *Pseudomonas aeruginosa* PAO1.

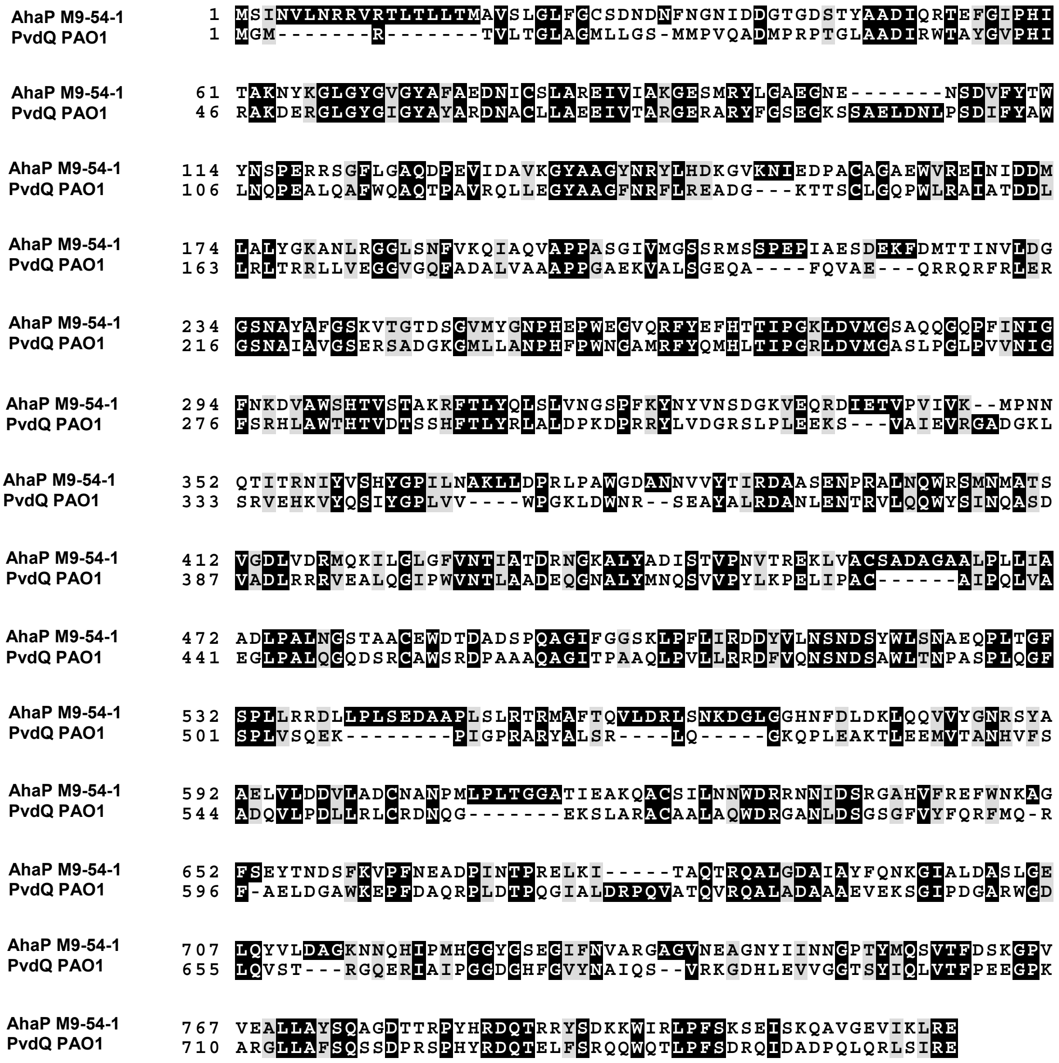

References

1. Dong, Y.H.; Xu, J.L.; Li, X.Z.; Zhang, L.H. AiiA, an enzyme that inactivates the acylhomoserine lactone quorum-sensing signal and attenuates the virulence of *Erwinia carotovora*. *Proc. Natl. Acad. Sci. U. S. A.* **2000**, *97*, 3526–31, doi:10.1073/pnas.060023897.
2. Morohoshi, T.; Nakazawa, S.; Ebata, A.; Kato, N.; Ikeda, T. Identification and Characterization of N -Acylhomoserine Lactone-Acylase from the Fish Intestinal *Shewanella sp.* Strain MIB015. *Biosci. Biotechnol. Biochem.* **2008**, *72*, 1887–1893, doi:10.1271/bbb.80139.
3. Chen, C.-N.; Chen, C.-J.; Liao, C.-T.; Lee, C.-Y. A probable aculeacin A acylase from the *Ralstonia solanacearum* GMI1000 is N-acyl-homoserine lactone acylase with quorum-quenching activity. *BMC Microbiol.* **2009**, *9*, 89, doi:10.1186/1471-2180-9-89.
4. Park, S.Y.; Lee, S.J.; Oh, T.K.; Oh, J.W.; Koo, B.T.; Yum, D.Y.; Lee, J.K. AhlD, an N-acylhomoserine lactonase in *Arthrobacter sp.*, and predicted homologues in other bacteria. *Microbiology* **2003**, *149*, 1541–1550, doi:10.1099/mic.0.26269-0.
5. Park, S.; Kang, H.; Jang, H.; Koo, B.; Yum, D.; Park, S.; Kang, H.; Jang, H.; Lee, J.; Koo, B. Identification of Extracellular N -Acylhomoserine Lactone Acylase from a *Streptomyces* sp. and Its Application to Quorum Quenching. *Appl. Environ. Microbiol.* **2005**, *71*, 2632–2641, doi:10.1128/AEM.71.5.2632.
6. Morohoshi, T.; Tominaga, Y.; Someya, N.; Ikeda, T. Complete genome sequence and characterization of the N-acylhomoserine lactone-degrading gene of the potato leaf-associated *Solibacillus silvestris*. *J. Biosci. Bioeng.* **2012**, *113*, 20–25, doi:10.1016/j.jbiosc.2011.09.006.

7. Wang, W.Z.; Morohoshi, T.; Someya, N.; Ikeda, T. Aidc, a Novel N-Acylhomoserine Lactonase from the Potato Root-Associated *Cytophaga-Flavobacteria-Bacteroides* (CFB) Group Bacterium *Chryseobacterium sp.* Strain strb126. *Appl. Environ. Microbiol.* **2012**, *78*, 7985–7992, doi:10.1128/AEM.02188-12.
8. Czajkowski, R.; Krzyzanowska, D.; Karczewska, J.; Atkinson, S.; Przysowa, J.; Lojkowska, E.; Williams, P.; Jafra, S. Inactivation of AHLs by *Ochrobactrum* sp. A44 depends on the activity of a novel class of AHL acylase. *Environ. Microbiol. Rep.* **2011**, *3*, 59–68, doi:10.1111/j.1758-2229.2010.00188.x.
9. Zhang, H.-B.; Wang, L.-H.; Zhang, L.-H. Genetic control of quorum-sensing signal turnover in *Agrobacterium tumefaciens*. *Proc. Natl. Acad. Sci. U. S. A.* **2002**, *99*, 4638–43, doi:10.1073/pnas.022056699.
10. Schipper, C.; Hornung, C.; Bijtenhoorn, P.; Quitschau, M.; Grond, S.; Streit, W.-R. Metagenome-derived clones encoding two novel lactonase family proteins involved in biofilm inhibition in *Pseudomonas aeruginosa*. *Appl. Environ. Microbiol.* **2009**, *75*, 224–233, doi:10.1128/AEM.01389-08.
11. Bijtenhoorn, P.; Schipper, C.; Hornung, C.; Quitschau, M.; Grond, S.; Weiland, N.; Streit, W.R. BpiB05, a novel metagenome-derived hydrolase acting on N-acylhomoserine lactones. *J. Biotechnol.* **2011**, *155*, 86–94, doi:10.1016/j.jbiotec.2010.12.016.
12. Shepherd, R.W.; Lindow, S.E. Two dissimilar N-acyl-homoserine lactone acylases of *Pseudomonas syringae* influence colony and biofilm Morphology. *Appl. Environ. Microbiol.* **2009**, *75*, 45–53, doi:10.1128/AEM.01723-08.
13. Jeng, Y.C.; Wu, L.; Wen, S.Y. Directed evolution of a quorum-quenching lactonase from *Mycobacterium avium* subsp. paratuberculosis K-10 in the amidohydrolase superfamily. *Biochemistry* **2009**, *48*, 4344–4353, doi:10.1021/bi9004045.
14. Bar-Rogovsky, H.; Hugenmatter, A.; Tawfik, D.S. The evolutionary origins of detoxifying enzymes: The mammalian serum paraoxonases (PONs) relate to bacterial homoserine lactonases. *J. Biol. Chem.* **2013**, *288*, 23914–23927, doi:10.1074/jbc.M112.427922.
15. Afriat, L.; Roodveldt, C.; Manco, G.; Tawfik, D.S. The latent promiscuity of newly identified microbial lactonases is linked to a recently diverged phosphotriesterase. *Biochemistry* **2006**, *45*, 13677–13686, doi:10.1021/bi061268r.
16. Huang, J.J.; Han, J.; Zhang, L.; Leadbetter, J.R. Utilization of Acyl-Homoserine Lactone Quorum Signals for Growth by a Soil Pseudomonad and *Pseudomonas aeruginosa* PAO1. *Appl. Environ. Microbiol.* **2003**, *69*, 5941–5949, doi:10.1128/AEM.69.10.5941.
17. Riaz, K.; Elmerich, C.; Moreira, D.; Raffoux, A.; Dessaux, Y.; Faure, D. A metagenomic analysis of soil bacteria extends the diversity of quorum-quenching lactonases. *Environ. Microbiol.* **2008**, *10*, 560–570, doi:10.1111/j.1462-2920.2007.01475.x.
18. Tannières, M.; Beury-Cirou, A.; Vigouroux, A.; Mondy, S.; Pellissier, F.; Dessaux, Y.; Faure, D. A Metagenomic Study Highlights Phylogenetic Proximity of Quorum-Quenching and Xenobiotic-Degrading Amidases of the AS-Family. *PLoS One* **2013**, *8*, doi:10.1371/journal.pone.0065473.
19. Huang, W.; Lin, Y.; Yi, S.; Liu, P.; Shen, J.; Shao, Z.; Liu, Z. QsdH, a Novel AHL Lactonase in the RND-Type Inner Membrane of Marine *Pseudoalteromonas byunsanensis* Strain 1A01261. *PLoS One* **2012**, *7*, 1–12, doi:10.1371/journal.pone.0046587.
20. Huang, J.J.; Petersen, A.; Whiteley, M.; Leadbetter, J.R. Identification of QuiP, the product of gene PA1032, as the second acyl-homoserine lactone acylase of *Pseudomonas aeruginosa* PAO1. *Appl. Environ. Microbiol.* **2006**, *72*, 1190–1197, doi:10.1128/AEM.72.2.1190-1197.2006.
21. Hiblot, J.; Gotthard, G.; Chabriere, E.; Elias, M. Structural and Enzymatic characterization of the lactonase SisLac from *Sulfolobus islandicus*. *PLoS One* **2012**, *7*, doi:10.1371/journal.pone.0047028.
22. Hiblot, J.; Gotthard, G.; Elias, M.; Chabriere, E. Differential Active Site Loop Conformations Mediate Promiscuous Activities in the Lactonase SsoPox. *PLoS One* **2013**, *8*, 1–14, doi:10.1371/journal.pone.0075272.
